# Identify Changes of Brain Regional Homogeneity in Bipolar Disorder and Unipolar Depression Using Resting-State fMRI

**DOI:** 10.1371/journal.pone.0079999

**Published:** 2013-12-04

**Authors:** Min-Jie Liang, Quan Zhou, Kan-Rong Yang, Xiao-Ling Yang, Jin Fang, Wen-Li Chen, Zheng Huang

**Affiliations:** 1 Medical Imaging Center, First Affiliated Hospital of Jinan University, Guangzhou, Guangdong, China; 2 MOE Key Laboratory of Laser Life Science & Institute of Laser Life Science, South China Normal University, Guangzhou, Guangdong, China; 3 Department of Electronic Engineering and CAPT Laboratory, University of Colorado Denver, Aurora, Colorado, United States of America; Banner Alzheimer's Institute, United States of America

## Abstract

**Background:**

To identify changes in brain activation patterns in bipolar disorder (BD) and unipolar depression (UD) patients.

**Methodology/Principal Findings:**

Resting-state fMRI scans of 16 healthy controls, 17 BD and 16 UD patients were obtained. *T-test* of normalized regional homogeneity (ReHo) was performed in a voxel-by-voxel manner. A combined threshold of á = 0.05, minimum cluster volume of *V* = 10503 mm^3^ (389 voxels) were used to determine ReHo differences between groups. In UD group, fMRI revealed ReHo increases in the left middle occipital lobe, right inferior parietal lobule, right precuneus and left convolution; and ReHo decreases in the left parahippocampalgyrus, right precentralgyrus, left postcentralgyrus, left precentralgyrus and left cingulated. In BD group, ReHo increases in the right insular cortex, left middle frontal gyrus, left precuneus, left occipital lobe, left parietal, left superior frontal gyrus and left thalamus; and ReHo decreases in the right anterior lobe of cerebellum, pons, right precentralgyrus, left postcentralgyrus, left inferior frontal gyrus, and right cingulate. There were some overlaps in ReHo profiles between UD and BD groups, but a marked difference was seen in the thalamus of BD.

**Conclusions/Significance:**

The resting-state fMRI and ReHo mapping are a promising tool to assist the detection of functional deficits and distinguish clinical and pathophysiological signs of BD and UD.

## Introduction

Bipolar disorder (BD) is a common psychiatric condition and the sixth leading cause of disability that affects 1.5–3.0% of the population worldwide [1], [2]. BD can cause various degrees of manic, hypomanic and depressive states. According to the Diagnostic and Statistical Manual of Mental Disorders (DSM-IV) it might be easy to make diagnosis when BD patient is in the manic and hypomanic state [3], but BD patients who are mainly in depressive episodes without a history of mania are often misdiagnosed [4]. Consequently, these patients are often treated as having recurrent unipolar depression (UD), which can lead to inadequate treatment, increased medical costs and poor outcomes [5]–[7]. Therefore, it is critical for psychiatrists to make correct diagnosis and initiate proper treatment [8]. Brain imaging techniques might offer a useful tool to reveal some critical changes in the brain of BD patients and assist in the diagnosis of BD.

Magnetic resonance imaging (MRI), positron emission tomography (PET), magnetic resonance spectroscopy (MRS) and functional MRI (fMRI) have been used to study the neural status in depression. For instance, structural MRI can reveal abnormalities in various brain areas and changes in the amygdala volume in adults suffering from depression and mood disorders [9]–[11]. Task-state fMRI shows that BD may arise from abnormalities within discrete brain networks [12]. The diminished prefrontal modulation of subcortical and medial temporal structures within the anterior limbic network (e.g. amygdala, anterior striatum and thalamus) can result in the deregulation of mood [13]. In particular, the resting-state fMRI has become an attractive tool to study resting-state brain function and mood disorder [14]. The non-invasive resting-state fMRI is easy to apply without a need of complicated task design, thus making it readily accepted by psychiatric patients. Because neither stimulation nor response is required, the resting-state fMRI has received increasing attention for studying BD and UD [15], [16]. Some investigators believe that the resting brain functional status is as important as the activity-evoked status [17]. During rest, the brain exhibits a functional architecture that includes both “task-negative” and “task-positive” networks [18]. A recent study indicated that there was a link between the decrease of corticolimbic functional connectivity and mood disorders [19].

Currently, the resting state data processing methods include seed-based approaches, amplitude of low frequency fluctuation (ALFF) and fractional amplitude of low-frequency fluctuation (fALFF) independent component analysis (ICA), regional homogeneity (ReHo) analysis, and so on [20]. Hypothesis-driven seed-based analysis is simple, sensitive and easy to interpret method. It is favored in revealing the intermittent interactions between spatially distinct brain regions due to its relatively for interpreting the dates [21]. A priori selection of the seed region is required before conduction the study, and this method is to investigate the degree of correlation among Regions of interest (ROI) or between ROI and whole brain voxels, to determine whether they have higher similarity between these brain areas and ROI in function (the Functional Connectivity) [22], [23]. Data-driven ICA is data analysis method to find the unknown variables or components from multivariate statistical data. It was applied for the first time in fMRI data analysis by McKeown MJ [24]. The difference between the ICA and other methods is that it can effectively separate the various function signal of independent statistical data and all kinds of noise without any a priori assumptions about times [25]. However, these two methods do not provide direct information regarding regional brain features.

As an alternative, the local brain activity information can be assessed by the ALFF, fALFF or ReHo. The method of ALFF assumed that the BOLD signal of resting brain have physiological significance in the low frequency range (e.g. 0.01 to 0.08 Hz), and to use an average amplitude of all frequencies between low frequency band to describe the intensity of a voxel spontaneous activity, and to reflect the intensity of regional spontaneous brain activity in resting state from the energy point of view [26]. As a normalized index of ALFF, The fALFF is concerned with the ratio of power spectrum of low-frequency (e.g. 0.01 to 0.08 Hz) to that of the entire frequency range (e.g. 0 to 0.25 Hz) [27]. Compared with ALFF, the fALFF can improved the sensitivity and specificity to reflect intrinsic neuronal activity and physiological states within specific regions in detecting spontaneous brain activities [27], [28]. Moreover, the fALFF can provide a more specific index of low frequency oscillatory phenomena in consideration of that the total power over the entire detectable frequency ranges present in the BOLD signal.

We used ReHo analysis to processed data in our study. ReHo analysis was originally proposed for measuring the degree of regional synchronization of fMRI time courses. Since the brain acts in the form of a mass or region of many cluster volumes, the analysis of the regional brain activity such as ReHo can be a useful means to evaluate the similarity of brain activity between the adjacent cluster volumes [29]. The consistency of a conglomeration (in time series), which consists of a given cluster and its adjacent clusters in the whole brain, can be assessed by the ReHo method since it can be assumed that the hemodynamic characteristics of every voxel are similar within a functional cluster and there is a dynamic synchronization of voxels within a given cluster, therefore reflecting the temporal homogeneity of the regional blood oxygen level-dependent (BOLD) signal (rather than its density and signal intensity) and the homogeneous condition of the regional brain region and time series indirectly. Abnormal ReHo is likely related to changes in the temporal aspects of spontaneous neural activity in the regional brain [29]. Therefore, an abnormal ReHo (either increase or decrease) may be a clue that is likely related to unbalanced local functionality or to an uncompensatory reaction of the whole brain network.

The ReHo analysis has been used to study Alzheimer's disease [30], [31], Parkinson's disease [32], [33], pediatric epilepsy [34], Schizophrenia [35], BD [36], [37] and UD [38]. The analysis of regional ReHo change has also be tested for differential diagnosis of BD and UD [15]. Based on available data, we hypothesize that ReHo profiles in BD (under depressive state) and UD might be different and such differences can be detected by the resting-state fMRI. In this study, for the first time, the ReHo variation analysis of the whole brain of BD patients under depressive state was carried out on the resting-state fMRI to identify differences of regional spontaneous activity in the whole brain between normal subjects, BD patients and UD patients.

## Materials and Methods

### 1. Subjects

The protocol for this trial and CONSORT checklist S1 are available as supporting information; see Checklist S1. Initially a total of 16 healthy subjects (control), 18 UD patients and 20 BD (under depressive state) patients were recruited for this study. All of them were right-handed native Chinese speakers and medication-free. UD and BD patients were recruited from the Outpatient Clinic of First Affiliated Hospital of Jinan University. Gender, age and education matched healthy subjects were recruited from the local community through advertisements (December 2010 to January 2012). All subjects voluntarily signed the informed consent form. The study was approved by the Human Research Ethics Committee of Jinan University, Guangzhou China.

The clinical states of the patients were evaluated with the Hamilton anxiety scale (HAMA) and the 17-item Hamilton Depression Rating Scale (HAMD). Inclusion criteria for UD patients were as follows: 18–55 years old, able to give voluntary informed consent, met Diagnostic and Statistical Manual (DSM-IV) criteria for Major Depressive Episode (i.e. the score of a 17-item HAMD ≥17) [39], qualified to undergo MRI scans, and able to be managed as an outpatient. Inclusion criteria for BD patients were as follows: met the DSM-IV criteria for BD, currently in the midst of a depressive episode, Young Mania Rating Scale (YMRS) <7, and 17-item HAMD score ≥17 [40]. Exclusion criteria were as follows: met DSM-IV criteria for schizophrenia, schizoaffective disorder, or an anxiety disorder as a primary diagnosis, use of psychotropic agents in the past 2 weeks, use of fluoxetine in the past 4 weeks, met DSM-IV criteria for substance dependence within the past year, use of caffeine or nicotine, positive urinary toxicology screening, use of alcohol in the past week, serious medical or neurological illness, pregnant or breast-feeding, and having metallic implants or other contraindications to MRI scan.

Inclusion criteria for healthy subjects were as follows: 18–55 years old, able to give voluntary informed consent, no history of psychiatric illness or substance abuse or dependence, no significant family history of psychiatric or neurological illness, not currently taking any prescriptions, no use of alcohol in the past week, and no serious medical or neurological illness. Exclusion criteria for healthy subjects were as follows: pregnant, breast-feeding, and metallic implants or other contraindication to MRI scan.

### 2. Behavioral ratings

Subjects were rated by the 17-item HAMD and the HAMA at the time of the baseline MRI scans.

### 3. MRI scans

MRI scans were performed on a 1.5 Tesla MR system (GE Signa HD MR) in the afternoon or the early evening. Foam pads were used to limit head motion. During scanning, participants were explicitly instructed to keep their eyes closed, relax, move as little as possible, think of nothing in particular but not fall asleep. Three-dimensional T1-weighted (3D-T1W) images were acquired in a sagittal orientation by employing a 3D-SPGR sequence (TR/TE = 18/1.8 ms, flip angle = 20°, in-plane resolution of 256×256, 1.8 mm slice thickness). Functional images were collected using a gradient Echo Planar Imaging (EPI) sequence sensitive to BOLD contrast (TR/TE = 3000/60 ms, flip angle = 90°, FOV = 24 cm). Whole-brain volumes were acquired with 20 contiguous 5-mm thick transverse slices, with a 1 mm gap and 3.75 mm×3.75 mm in-plane resolution. The fMRI scanning lasted 6 min and 24 s to obtain 128 volumes on each subject. After scanning, the patient was interviewed to assess whether he or she complied with the instructions and were awake throughout the scan. Subjects who failed to comply with the instructions would be excluded from the study.

### 4. Image analysis

Imaging data were processed by MATLAB 2009b (Mathworks, Natick, MA, USA) and DPARSF (Data Processing Assistant for Resting-State fMRI) software [41] for DICOM transformation, slice timing, head motion correction, and spatial normalization. Motion time courses were obtained by estimating the values for translation (mm) and rotation (degrees) for each subject. The participants who had more than 1 mm maximum displacement in x, y, or z and 1° of angular motion during fMRI scans would be rejected. Functional data were evaluated by MRIcro software (http://www.mricro.com/) to detect defective data. The first 10 time points of the functional images were discarded due to the possible instability of the initial MRI signals and the participants' adaptation to the scanning environment. After head-motion correction, the fMRI images were normalized to the Montreal Neurological Institute (MNI) template with a re-sampling voxel volume of 3 mm×3 mm×3 mm. The 3D-T1W images were also spatially normalized to the MNI template. Functional images were smoothed with a Gaussian filter of 8 mm×8 mm×8 mm full-width at half maximum (FWHM). After pre-processing, the time series for each voxel was detrended and filtered (using the typical frequency band 0.01–0.08 Hz) to reduce low-frequency drift, physiological high frequency respiratory and cardiac noise, and time series linear detrending. A mask was then created by taking the intersections of the normalized T1-weighted high-resolution images of all subjects, which were stripped using the BrainSuite software (http://brainsuite.usc.edu). Only the voxels within the mask were further processed. In addition, in order to visualize the statistical results, a mean anatomical image was obtained by averaging normalized high-resolution anatomical images across all subjects.

ReHo analysis was performed with the REST software (http://www.Resting- fmri.Sourceforge.net). A full factorial model in SPM8 was implemented to regress out nuisance covariates with factors such as age, gender, years of education, and HAMA score. Individual ReHo mappings were generated by calculating the Kendall's coefficient concordance (KCC) of the time series of a given voxel with those of its nearest neighbors [42]. In previous studies [43], [44], the KCC (ReHo value) was standardized by being divided by the global mean. However, it has been recently suggested that transformation into standard Z value (i.e. subtracting the global mean, then being divided by standard deviation) could improve the normality of distribution [45]. The number of neighboring voxels was set as 26. To reduce the influence of individual variations, the normalization of ReHo mapping was carried out by dividing the KCC among each voxel by the average KCC of the whole brain for each subject [32], [46].

The consistency and similarity for each individual were assessed by calculating the KCC of the time series of one given voxel with those of its adjacent voxels in a voxel-wise analysis assuming that a voxel was temporally similar to those of its neighbors [47]. The computational formula for calculating the KCC value has been explained in previous studies [29], [48] and calculated as follows:
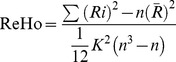
Where ReHo is the KCC among given voxels, ranging from 0 to 1. When a given cluster and its adjacent cluster in a time series is more consistent, the KCC value is more close to 1. *K* is the voxel number of time series within a measured cluster (the smallest unit of measured ReHo, composed of more adjacent clusters); here, *K* = 27 (one given voxel which was located in the cubic center plus its adjacent 26 voxels). n is the number of ranks; here, *n* = 128. Ri is the sum rank of the i^th^ time point, where 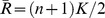 is the mean of the Ri's. Standard ReHo value is the ReHo value of each cluster/the mean of the whole brain ReHo value. Ultimately, the individual ReHo map was generated for each data set.

### 5. Statistical analysis

Chi-square test was used for gender comparison. ANOVA tests were performed to identify the differences in age, education and neuropsychological test comparisons between BD (under depressive state), UD and control groups. Bonferroni multiple comparison correction tests (at p<0.05) were used for post-hoc analyses of paired comparisons when those data show significant effects in this ANOVA. Two sample t-tests were performed to examine the voxel-wise difference on the ReHo maps between the BD, UD and control groups. The cluster extent threshold *V*>10503 mm^3^ (389 voxels) and *T*>2.042 were used to determine statistical significance, which means *P*<0.05 for each voxel. For multiple comparison correction, this combined threshold was determined by Monte Carlo simulation with AlphaSim command.

## Results

### 1. Group characteristics and psychological data

Due to excessive head movement during scanning, imaging data from 2 UD and 3 BD patients were excluded and consequently a total of 49 participants were included in this study. Their demographic data and psychological scores are listed in Table 1. An ANOVA showed there were no significant differences between BD, UD, and health control groups in terms of educational level (F = 0.327, P = 0.723) and age (F = 0.128, P = 0.881). Two-sample t-tests showed there were significant differences in terms of the HAMD and HAMA Rating Scales between controls and BD patients (t_HAMA_ = 6.674, P = 0.015; t_HAMD_ = 22.130, P = 0.000) or UD patients (t_HAMA_ = 6.611, P = 0.015; t_HAMD_ = 12.514, P = 0.001), but no significant difference between BD and UD patients (t_HAMA_ = 0.001, P = 0.980; t_HAMD_ = 0.043, P = 0.837). Chi-square test showed there were no significant differences between the three groups in terms of gender distribution (χ^2^ = 1.200, P = 0.273).

**Table 1:** Demographic and psychological data of UD patients, BD patients and controls.

	UD	BD	Control	P- value	
No. of subjects	16	17	16		
Age, years	36.06±9.43	34.47±9.77	35.13±7.88	0.88	a
Age range	20–53	19–53	23–50		
Education, years	12.75±2.82	12.06±2.90	12.75±2.82	0.72	a
Gender (M/F)	8/8	9/8	8/8	0.27	b
HAMA score	18.19±2.34	18.41±2.32	2.75±1.13		c
HAMD score	26.19±4.98	24.47±4.87	4.38±1.63		c
Duration of illness, years	2.36±1.89	3.89±3.56		0.35	d
Depressive episodes number	3.08±1.45	8.23±6.94		0.57	d

Bold data indicate significance level at p<0.05.

Abbreviations: HAMD Scales: the Hamilton Depression Rating Scales; HAMA Scales: Hamilton Anxiety Rating Scales.

a : Indicates p-values for one-way ANOVA.

b : Indicates p-values for Chi-square tests.

c : Indicates p-values for ANOVA and post-hoc t-tests.

d : Indicates p-values for two-sample t-tests.

(Mean ± S.D.).

### 2. ReHo profiles

**Figure 1:**
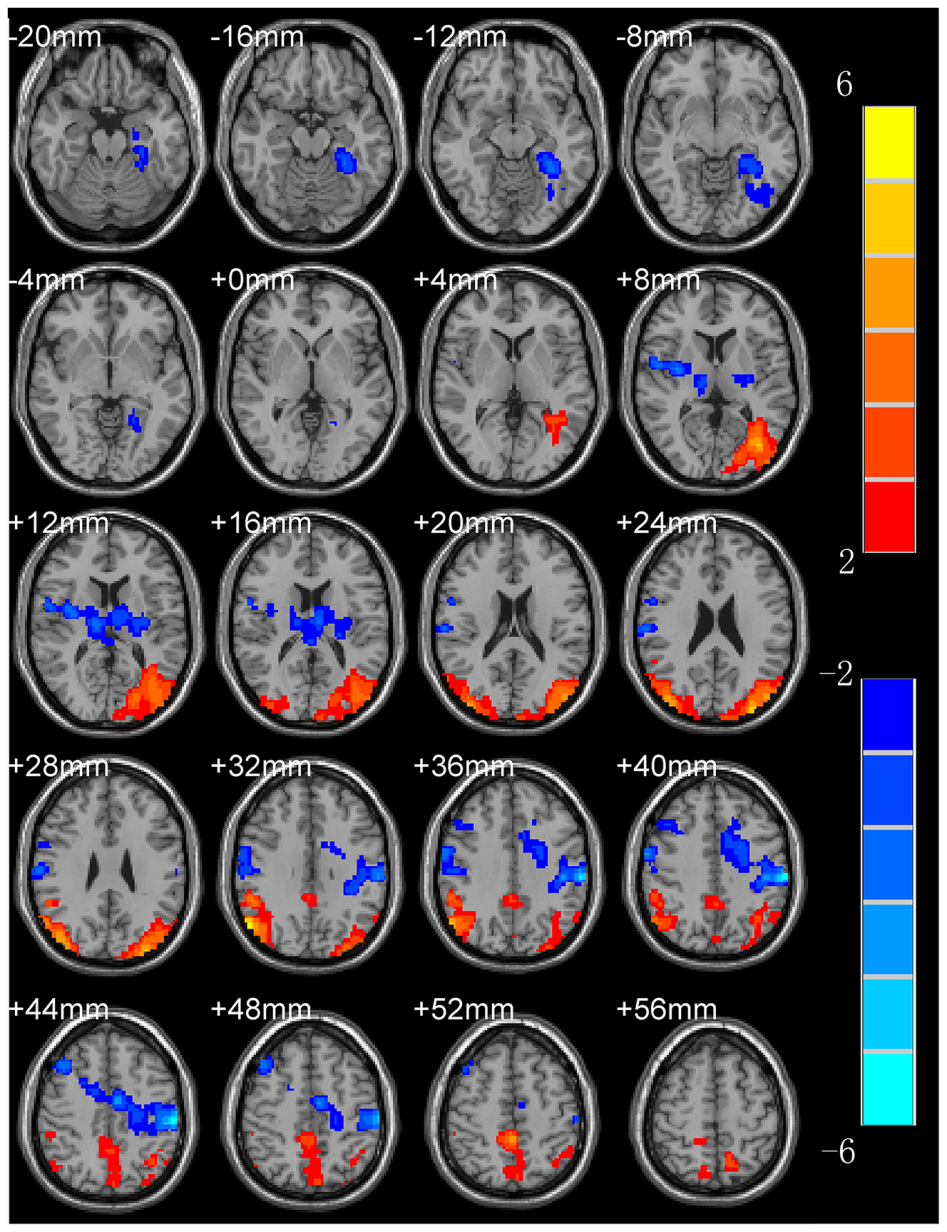
ReHo mapping of UD patients (compared to controls). Hot and cold colors indicate the increase and decrease of ReHo, respectively. Numbers in the upper left of each image refer to the *z*-plane coordinates of the MNI space.

ReHo maps of UD patients and controls are shown in Table 2 and Fig. 1. The results obtained from the two-sample *T*-test clearly demonstrated there were significant differences between the two groups. Compared to the controls, UD patients showed a significant decrease of ReHo in the left parahippocampalgyrus, the right precentralgyrus, the left postcentralgyrus, the left precentralgyrus and left cingulated, but significant increase of ReHo in the left middle occipital lobe, right inferior parietal lobule, right precuneus and left convolution.

**Table 2:** Regions with increased and decreased ReHo in UD patients compared to controls.

Anatomical region	BA	Cluster Size (Voxels)	*T* a	MNI^b^		
				X	Y	Z
L. middle occipital lobe	19	408	3.921	−33	−85	27
R. inferior parietal lobule	40	636	5.836	57	−69	33
R. precuneus	23	455	4.342	0	−42	51
L. convolution	39	422	3.501	−43	−77	27
L. parahippocampalgyrus	28	466	−3.994	−15	6	−33
R. precentralgyrus	4	655	−3.818	63	−3	39
L postcentralgyrus	2	860	−4.788	−57	−25	39
L. precentralgyrus	6	540	−3.349	−57	−17	39
L. cingulated	46	485	−2.938	−24	−4	39

NB: BA = Brodmann's area, R = right, L = left.

Positive sign represents regions with increase ReHo, and negative sign represents regions with decrease ReHo.

a Represents the statistical value of peak voxel showing ReHo differences comparing UD patients and controls. Positive *T* value indicates the increase of ReHo, and negative *T* value indicates the decrease of ReHo, respectively.

b Coordinates of primary peak locations in the MNI space.

**Figure 2:**
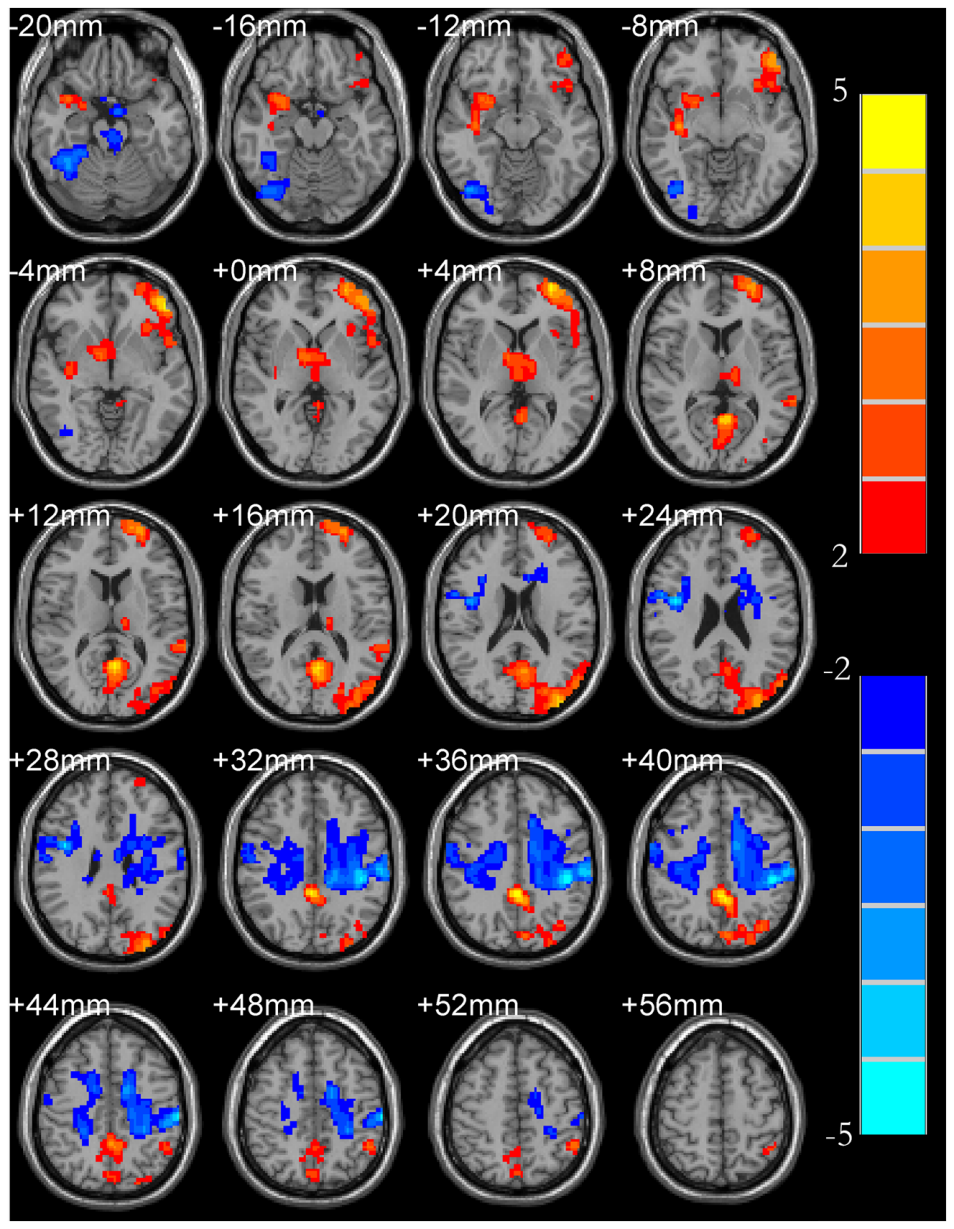
ReHo mapping of BD (under depressive state) patients (compared to controls). Hot and cold colors indicate the increase and decrease of ReHo, respectively. Numbers in the upper left of each image refer to the *z*-plane coordinates of the MNI space.

ReHo profiles of BD patients are shown in Table 3 and Figure 2. The results obtained from the two-sample t-test clearly showed significant differences between BD and controls. Compared to the control, BD patients showed a significant decrease of ReHo in the right anterior lobe of cerebellum, pons, right precentralgyrus, left postcentralgyrus, left inferior frontal gyrus and right cingulate, but significant increase of ReHo in the right insular cortex, left middle frontal gyrus, left precuneus, left occipital lobe, left parietal, left superior frontal gyrus, and left thalamus.

**Table 3:** Regions with increased and decreased ReHo in BD patients compared to controls.

Anatomical region	BA	Cluster Size (Voxels)	*T* a	MNI^b^		
				X	Y	Z
R. insular cortex	13	581	3.498	39	−12	−9
L. middle frontal gyrus	6	812	4.882	−48	−45	−6
L. precuneus	23	1578	4.862	3	−42	36
L. parietal lobe	7	462	2.794	−5	−77	42
L. occipital lobe	19	438	2.683	−36	−77	42
L. superior frontal gyrus	11	422	3.836	−27	63	10
L. thalamus		415	2.651	−13	−13	10
R. anterior lobe of cerebellum	37	775	−4.136	27	−36	−24
pons		488	−4.208	−6	−12	−39
R. precentralgyrus	4	1061	−4.746	42	0	24
L. postcentralgyrus	2	1859	−4.877	−45	−30	36
L. inferior frontal gyrus	47	498	−2.516	33	13	24
R. cingulate	46	471	−2.435	−20	26	24

NB: BA = Brodmann's area, R = right, L = left.

Positive sign represents regions with increase ReHo, and negative sign represents regions with decrease ReHo.

a Represents the statistical value of peak voxel showing ReHo differences comparing BD patients and controls. Positive *T* value indicates the increase of ReHo, and negative *T* value indicates the decrease of ReHo, respectively.

b Coordinates of primary peak locations in the MNI space.

**Figure 3:**
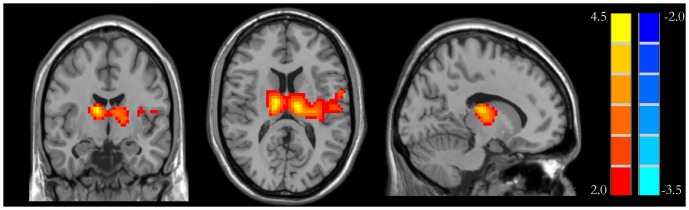
ReHo mapping of BD (under depressive state) patients (compared to UD patients) shows a significantly increase in ReHo in the thalamus. Hot and cold colors indicate the increase and decrease of ReHo, respectively.

Comparisons of ReHo between BD and UD patients are listed in Table 4. The two-sample t-test clearly showed significant differences between the two groups. In BD, there is a significant increase in ReHo in the thalamus, which is not present in the UD; and this difference is also significant. The BD group also showed a significantly higher ReHo in the thalamus (Fig. 3).

**Table 4:** Comparison of ReHo in the thalamus in BD and UD patients.

Anatomical region	Cluster Size (Voxels)	*T* a	MNI^b^		
			X	Y	Z
Thalamus	605	4.343	12	−12	15

Positive sign represents regions with increase ReHo.

a Represents the statistical value of peak voxel showing ReHo differences comparing UD and BD patients. Positive *T* value indicates higher ReHo in BD patients.

b Coordinates of primary peak locations in the MNI space.

## Discussion

To the best of our knowledge, there are few studies that visualize the whole brain ReHo profiles of BD and UD patients during brain spontaneous activity using fMRI. The analyses of resting-state functional connectivity have been used to investigate the default-mode network of the brain. It has been demonstrated that in the absence of any stimulus the network exhibits temporally coherent low-frequency fluctuations of the BOLD signal [49], [50]. However, such functional connectivity studies need to select a ROI for examining relationships among connected regions. In contrast, the ReHo method used in this study does not need to utilize ROIs and therefore can avoid possible variations associated with ROI selection.

Abnormal frontal lobe, parietal lobe and occipital lobe may be related to depression in BD and UD. Our analysis of ReHo profiles in BD and UD showed that they shared some similarities in ReHo abnormalities in the frontal lobe, parietal lobe and occipital lobe. Meanwhile, each also showed some unique changes in the front lobe. In the UD group, there were decreases in the bilateral precentralgyrus and left cingulated, whereas in the BD group, there were decreases in the right precentralgyrus, right cingulated, and left inferior frontal gyrus (see Table 2, Table 3 and Figure 1, Figure 2).

The frontal lobe is the advanced cognitive center; it participates in integrating emotion and information about internal and external environment, and extracting episodic memory [51] in the resting-state. Depressive disorder patients have affective disorder meanwhile accompanied with persistent cognitive dysfunction, mainly demonstrated in executive dysfunction and memory impairment [52], [53]. Typical depression symptoms include depression, slow thinking and the psychokinesis hypoactivity, and the generations of these symptoms are associated with frontal lobe damage. According to the results of our experiment, it can be speculated that BD and UD patients show abnormal neural pathways with the frontal lobe as the center, thus leading to the abnormalities of executive function and emotional behaviour. Depression patients have clear suicidal tendencies, and BD are more severe than UD patients [54], probably related to the emotional damage of the frontal lobe. Experimental results showed that BD patients have higher ReHo value in left superior frontal gyrus and middle frontal gyrus (see Table 3 and Figure 2). The possible reason is that BD patients usually have more severe symptoms and larger area of abnormal ReHo value in frontal lobe than UD patients.

Cingulate cortex is an important part of the limbic system. The impairment of anterior cingulate cortex (ACC) will produce emotional instability, regulation dysfunction of autonomic nerve, apathy, akineticmutism and inattention in a series of clinical symptoms [55]. Posterior cingulated cortexes (PCC) are closely related to learning, memory, reward and punishment and executive function. There are many studies showing that the PCC can affect the behavior function through changing the decision-making and motivation [56]. The study found that BD and UD patients had abnormal ReHo value of cingulate cortex (see Table 2, Table 3 and Figure 1, Figure 2), which can be inferred that there is an abnormality of the frontal lobe- limbic system pathways in depressive disorder patients. The advanced features of the limbic system involved in the brain are emotional, mental, memory and other higher nervous activity. These results closely correspond to the clinical manifestations of depression, such as unresponsive brain, black mood, decline of memory and attention, low work productivity and learning inefficiencies.

A previous PET study showed that the back side of the bilateral parietal blood flow will increase according with the information complexity [57], and while the subjects were adopting this information, the bilateral parietal blood flow will decline. The changes of regular pattern of parietal lobe blood flow suggest that it is probably involved in learning and information transmission [58]. Mayberg et al. [59] found that the inferior parietal lobules of metabolism are decreased with depression disorder patients. In this study, the ReHo value was increased in inferior parietal lobule with BD and UD patients (see Table 2, Table 3 and Figure 1, Figure 2), suggesting that patients with parietal cortex dysfunction probably lead to the ability of receiving new information and learning is decreased. Shippee et al. [60] found that depression patients had low level of education compared with normal subjects in a large sample size of the survey, which is probably related to learning disabilities with parietal dysfunction.

The precuneus is a more active cerebral area in resting-state, primarily involved in episodic memory, self-awareness and self-reflection [61]. Grimm's [62] study found that the signal of precuneus was reduced when giving the positive and negative emotional stimuli of self-awareness in severe depression disorder patients in the task-status, suggesting that the precuneus is associated with negative emotions. Our study found that the ReHo value of precuneus was increased in BD and UD patients (see Table 2, Table 3 and Figure 1, Figure 2). This result showed that depression disorder patients' clinical symptoms of self-awareness strengthen and repeated introspection was related to precuneus dysfunction.

There are both many identical and different brain regions in respective ReHo activation area in BD and UD patients, which can be used to distinguish the two groups. There is a connection between insular with many other structural areas, such as the amygdala, superior temporal gyrus, orbital frontal cortex and hippocampus. Studies have found that the insular cortex metabolism will enhance when remembering sadness in the normal person [63]. The metabolism enhances of the insular were found in depression disorder patients, and the degree of enhancement associated with irritability [64] and sleep disorders [65]. In this study, the ReHo value of right insular increased in BD patients (see Table 3 and Figure 2), and they were prone to atypical symptoms such as irritability and drowsiness, which not only can be used to assess the symptoms of anxiety, irritability and sleep disorders in BD patients, and but also to distinguish BD and UD.

As we all know, the cerebellum is used to regulate body balance and muscle tension. Sasayama et al. [66] found that BD and UD patients have manual motor dexterity impairment, but BD is more severe than UD patients. The ReHo value of the cerebellum anterior lobe on the right side emerged abnormalities with BD groups in our experiment (see Table 3 and Figure 2), and suggest that this group of patients have cerebellar dysfunction. There is no abnormal ReHo value of cerebellar with UD group, probably because UD patients have fewer symptoms. In addition, the cerebellum also accepts the information from temporal lobe, prefrontal lobe and cingulate gyrus; these cerebral regions are related to cognitive and emotional regulation [67]. Schmahmann et al. [68] proposed the concept of the cerebellar cognitive affective syndrome, which usually refers from the affective bluntness and depression to affective disorder, and finally appears in execution, visual, spatial and language dysfunction. Therefore, it is simplistic view that the role of the cerebellum is only to adjust the balance and muscle tension, it is also involved in cognitive and emotional regulation. This study found that BD patients have abnormal ReHo value of anterior lobe in the right cerebellum, illustrating that the cerebellum participates in cognitive function and emotional regulation.

Malhi et al. [69] study found that positive language can stimulate the pons and have significant activation with normal group, but the patients group does not appear to have activation with task-state fMRI. Therefore, it can be speculated that the pons is involved in the processing of positive affective content. The ReHo value of pons of BD patients was significantly decreased (see Table 3 and Figure 2) which illustrated above view compared with normal group in this study. The probable reason is that BD patients have longer duration in general, and the cerebral region of emotion regulation were significantly increased compared with UD.

Hippocampus, parahippocampalgyrus and amygdala are critical structures which participate in the process of human learning and memory. A previous study also suggested that depression patients had hippocampal function and structure abnormality [70]. The potential mechanism is neurons and nutritional factors of hippocampus suffer damage under depression stress state, and this damage gradually increased with the duration of the extension and the volume of hippocampus was decreased. Bearden et al. found that both BD and UD patients had memory function damage, but no significant difference in the degree of injury [71]. The ReHo value of left parahippocampalgyrus of UD patients was decreased shown in our experiment (see Table 2 and Figure 1), but BD group have no abnormalities, the specific reasons needing further research.

A recent resting-state fMRI study of social phobia patients (in hyperarousal state) showed that the ReHo value of left middle occipital gyrus was increased and that could affect the patient's social communication skills [72]. Our study showed that the ReHo value of left middle occipital gyrus was increased in UD patients (see Table 2 and Figure 1) and it can be speculated that UD patients have memory decline and social difficulties. But we did not find abnormalities in the ReHo value of the occipital lobe in BD patients. It might be explained as the UD patients only have depressive episodes, but the BD patients have both mania and depression states.

Lui et al. [73] found that the bilateral anterior frontal lobe-limbic system-hypothalamus loop functional connectivity was significantly decreased in refractory depression disorder patients, which can be speculated that the abnormalities of hypothalamus-cortical loop has a direct relationship with the occurrence of depression. There are wide range of nerve fibers connection between nuclei in the hypothalamus and cerebral cortex; hypothalamic dysfunction can cause the abnormalities of hypothalamus-cortical loop, playing a vital role in the occurrence of depression. The functional connectivity between hypothalamus and the seed point of bilateral caudate nucleus could be enhanced with geriatric depression disorders patients from the Kenny's research [74]. Peng et al. found that the ReHo value of left thalamus was decreased in major depressive patients [75]. There were similar observations in our research, for instance, the ReHo value of the left thalamus was decreased in UD group (see Table 4 and Figure 3). Because the nerve fibers in the hypothalamus connect with telencephalon, cerebellum, striatum and amygdale, they are associated with emotional expression. Therefore, the main clinical symptoms of depression are probably related with the abnormalities of hypothalamus in UD patients.

It is apparent that BD patients showed significantly decrease of ReHo in larger areas of white matter (see Table 3 and Figure 2). The reason is probably that BD patients also have abnormalities in some white matter regions. MacFall et al. report that the impairment of the white matter in the frontal medial orbital in gerontal depression patients and the extent of damage is closely related to the degree of depression [76].

Our ReHo results demonstrated a trend of their correlation with clinical symptoms and neurological anatomy features of these two mental disorders. Although UD and BD have many differences in terms of pathogenesis and clinical symptoms, differences in ReHo were mainly found in the thalamus between UD and BD under direct comparison. However, these two mental disorders showed many differences in ReHo of the cerebral regions compared to that of the normal group, which is consistent with the findings reported by Liu et al. [15], [16]. Because the thalamus plays a vital role in the connection of neural pathways, it can be speculated that these two mental disorders are different in terms of the pathogenesis of neural pathways, which also leads to different clinical manifestations.

In conclusion, the measurement of functional changes in the resting-state BOLD signal is useful for identifying brain regions displaying differences between UD and depressive state BD. ReHo mapping shows that the distribution of some abnormal activities is different between the two disorders. The resting-state fMRI and ReHo mapping is a promising tool to assist in the detection of functional deficits and distinguish clinical and pathophysiological signs of these two common mental disorders.

## Supporting Information

Consort Checklist S1 Checklist of information to include when reporting a randomised trial.
(DOC)
